# Case report of a child with nephronophthisis from South Africa

**DOI:** 10.1186/s12887-024-04872-2

**Published:** 2024-07-05

**Authors:** Rajendra Bhimma, Edgar Jembere, Sudesh Hariparshad

**Affiliations:** 1 Department of Paediatrics and Child Health, College of Health Sciences, Nelson R Mandela School of Medicine, University of KwaZulu-Natal, Durban, South Africa; 2 School of Mathematics, Statistics and Computer Science, University of KwaZulu-Natal, Durban, South Africa; 3 Present Address: Department of Nephrology, College of Health Sciences, Nelson R Mandela School of Medicine, University of KwaZulu-Natal, Durban, South Africa

**Keywords:** Nephronophthisis, Chronic kidney failure, Children, Genetic testing

## Abstract

**Background:**

Nephronophthisis (NPHP) is an autosomal recessive disorder with a subset of patients presenting with extrarenal manifestations such as retinal degeneration, cerebella ataxia, liver fibrosis, skeletal abnormalities, cardiac malformations, and lung bronchiectasis. However, the involvement of other organ systems has also been documented. Extrarenal manifestations occur in approximately 10–20% of patients. In developed countries, it has been reported as one of the most common causes of monogenic chronic kidney failure (CKF) during the first three decades of life, with more than 25 genes associated with this condition. The current treatment options for managing NPHP include supportive care, management of complications, and kidney replacement therapy when necessary.

The index patient is a 10-year-old Caucasian female who presented with recurrent attacks of abdominal pain. Her elder sister, TN, who was 17 years old, was diagnosed with CKF and noted to have persistently elevated liver enzymes (gamma-glutamyl transferase, alanine, and aspartate transaminases). Following genetic testing, her elder sister was shown to have Nephronophthisis Type 3, and a liver biopsy showed early fibrotic changes. Subsequent genetic testing confirmed the index patient as having NPHP Type 3. A kidney biopsy showed focal sclerosed glomeruli with patchy areas of tubular atrophy and related tubulointerstitial changes in keeping with NPHP. We present the first confirmatory case of NPHP from South Africa based on histopathology and genetic testing in a 10-year-old Caucasian female who presented with recurrent attacks of abdominal pain, whose elder sister also presented with CKF and early liver fibrosis, confirmed on biopsy and genetic testing.

**Conclusion:**

In low-middle-income countries, genetic testing should be undertaken whenever possible to confirm the diagnosis of NPHP, especially in those with a suggestive biopsy or if there is CKF of unknown aetiology with or without extra-renal manifestations.

## Introduction (background)

Nephronophthisis (NPHP) is an autosomal recessive disorder with a subset of patients presenting with extrarenal manifestations. These include retinal degeneration, cerebella ataxia, liver fibrosis, skeletal abnormalities, cardiac malformations, and lung bronchiectasis. Involvement of other organ systems has also been documented. Extrarenal manifestations occur in approximately 10–20% of patients [1]. NPHP has been reported as one of the most common causes of monogenic chronic kidney failure (CKF) during the first three decades of life, with more than 25 genes associated with this condition [2]. These genes encode proteins involved in the function of primary cilia, basal bodies, and centrosomes, resulting in kidney disease and extra-kidney manifestations [3–5]. Mutations in the *NPHP1* gene are the most common, accounting for around 20% of cases, with each remaining NPHP gene accounting for < 1% of cases and around two-thirds of cases remaining genetically unknown [6].

Three clinical variants of this condition are described based on the expected age for CKF viz. infantile (one year of age), juvenile (13 years of age), and adolescent (19 years of age). The rate of progression to CKF is determined in part by the type and severity of the genetic defect [7]. These variants and extrarenal organ abnormalities are usually associated with specific gene defects [8].

Clinical manifestations in childhood and adolescents include polydipsia and polyuria (due to the inability of the kidneys to concentrate solutes and impaired sodium reabsorption), secondary enuresis, symptoms of anaemia, and growth retardation. Urinalysis typically shows a “bland urine” without evidence of blood, protein, or cellular elements and mild tubular proteinuria in the early stages of the disease, not detected on routine dipstick analysis. It is only in the late stages of the disease that proteinuria may develop secondary to glomerulosclerosis. Poor growth may be initially related to chronic hypovolemia and later due to growth retardation secondary to chronic kidney disease. Blood pressure is usually normal, most likely due to hypovolemia caused by impaired urinary concentration and kidney salt wasting [9, 10]. Anaemia due to erythropoietin deficiency may be a presenting finding of tubulointerstitial involvement before a significant decline in kidney function [11, 12].

Imaging shows normal or slightly decreased-in-size kidneys with increased echogenicity, reduced corticomedullary differentiation, and renal cysts on ultrasound examination. If genetic testing is unavailable or the diagnosis remains uncertain, a kidney biopsy is performed in patients with a negative genetic test. The histopathological findings of chronic tubulointerstitial changes with irregular thickening of tubular basement membranes are typical of this disorder, and dilation of tubules with cyst formation mainly at the corticomedullary border [1].

There is a paucity of data on NPHP reported from the African continent. Senior et al. reported a family in Johannesburg, South Africa, with at least four, and probably six, of 13 children with nephropathy closely resembling that of juvenile-onset NPHP. All six patients had retinal dystrophy indistinguishable from Leber’s tapetoretinal degeneration. Genetic testing was unavailable, but the kidney biopsy on the index patient showed histopathology consistent with pan nephritis with arteriolosclerosis and closely resembled that of familial juvenile nephronophthisis [13]. A case of Schimke immuno-osseous dysplasia, steroid-resistant nephrotic syndrome, and Moyamoya syndrome was reported by Govender et al. from Durban, South Africa, with genetic studies that confirmed the presence of the c.1439 C > T mutation in the SMARCAL1 gene. Renal ultrasound demonstrated poor cortico-medullary differentiation, and the kidney biopsy showed features of focal segmental glomerulosclerosis with some interstitial changes, and the patient’s kidney function remained normal until she demised [14]. However, this patient did not have other features suggestive of NPHP on kidney biopsy. Genetic testing did not confirm NPHP.

This is the first case report of an adolescent presenting with clinical features, genetic mutations, and kidney histopathological findings consistent with NPHP reported in our region and Africa.

## Case report(presentation)

The index patient is a 10-year-old Caucasian female who presented with recurrent attacks of abdominal pain in 2017. As both her siblings had developed appendicitis at an early age, her house doctor did an abdominal ultrasound (US). Both kidneys were of average size, with no pathology noted except for being reported as echogenic. A repeat US for recurrent abdominal pain in 2018 showed the same findings.

In April 2018, her elder sister, who was 17 years old, was diagnosed with CKF and commenced haemodialysis. During routine laboratory testing, the elder sister was noted to have persistently elevated liver enzymes (gamma-glutamyl transferase, alanine, and aspartate transaminases). Following genetic testing, the elder sister was shown to have Nephronophthisis Type 3, and a liver biopsy showed early fibrotic changes. Their brother, 20 years old, was also screened for liver and kidney disease, but all tests were normal.

**Fig. 1:**
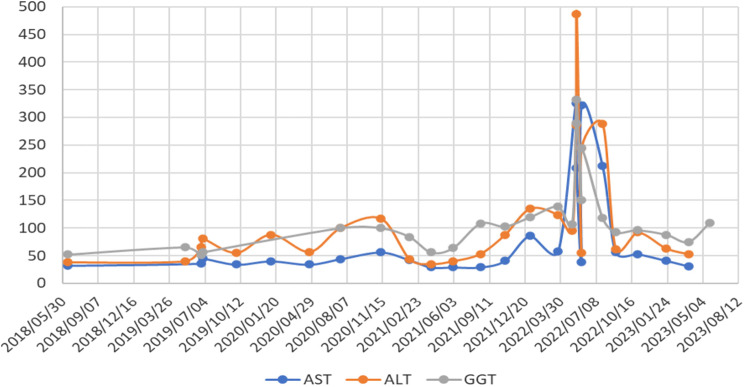
Elevation of liver enzymes (u/L)

A repeat US done on the index patient in early 2019 showed echogenic kidneys with normal architecture. Laboratory testing showed marginally raised liver enzymes (< 5 x normal) (Fig. 1). Subsequent genetic testing confirmed her as having NPHP Type 3 (Table 1).

**Table 1:** Pathogenic variants identified in *NPHP3*. Two Pathogenic variants were identified in NPHP3. NPHP3 is associated with a spectrum of autosomal recessive conditions involving ciliary dyskinesia

Additional Variant(s) of uncertain significance identified				
Gene	Variant	Zygosity	Variant classification	
NPHP3	c.2694-2_2694-1del (Splice site)	heterozygous	PATHOGENIC	
NPHP3	c.2154 C > T (Silent)	heterozygous	PATHOGENIC	
NPHP4	c.2207G > A (pArg736His)	heterozygous	Uncertain significance	
**Interpretation**				
Gene	Variant	Zygosity	Prior variant classification	New variant classification
NPHP3	c.2154 C > T (Silent)	heterozygous	Likely Benign	PATHOGENIC

This diagnostic test evaluated 27 gene(s) for variants (genetic changes) associated with genetic disorders

On subsequent visits, her liver enzymes showed an incremental rise; her cholesterol remained elevated, and a repeat US confirmed echogenic kidneys with normal size and architecture. In January 2020, she was referred to a gastroenterology team in another province. An endoscopic retrograde pancreatogram found non-specific changes without evidence of liver cirrhosis on liver biopsy. In June 2020, she showed a rising creatinine level, and she had, by the end of September 2022, progressed to CKD stage II, with an estimated glomerular filtration rate (eGFR) using the modified Swartz formula of 66% (Fig. 3). At this stage, she developed persistent headaches and episodes of nausea (no vomiting), but no symptoms of retinitis pigmentosa such as night blindness, loss of peripheral vision, tunnel vision or trouble seeing colours, and fundoscopic examination were normal. However, her abdominal pains persisted. She was diagnosed as having an abdominal migraine. Apart from these symptoms, she had no syndromic features or other extrarenal manifestations associated with nephronophthisis. A kidney biopsy in May 2022 showed focal sclerosed glomeruli with patchy areas of tubular atrophy and related tubulointerstitial changes. There was no evidence of any cystic changes at the corticomedullary junction or evidence of segmental sclerosis. The pathologist reported the kidney biopsy findings as consistent with nephronophthisis. She had an acute exacerbation of her abdominal pains associated with nausea, vomiting, and rigours, with rising liver enzymes (Fig. 1).

**Fig. 2:**
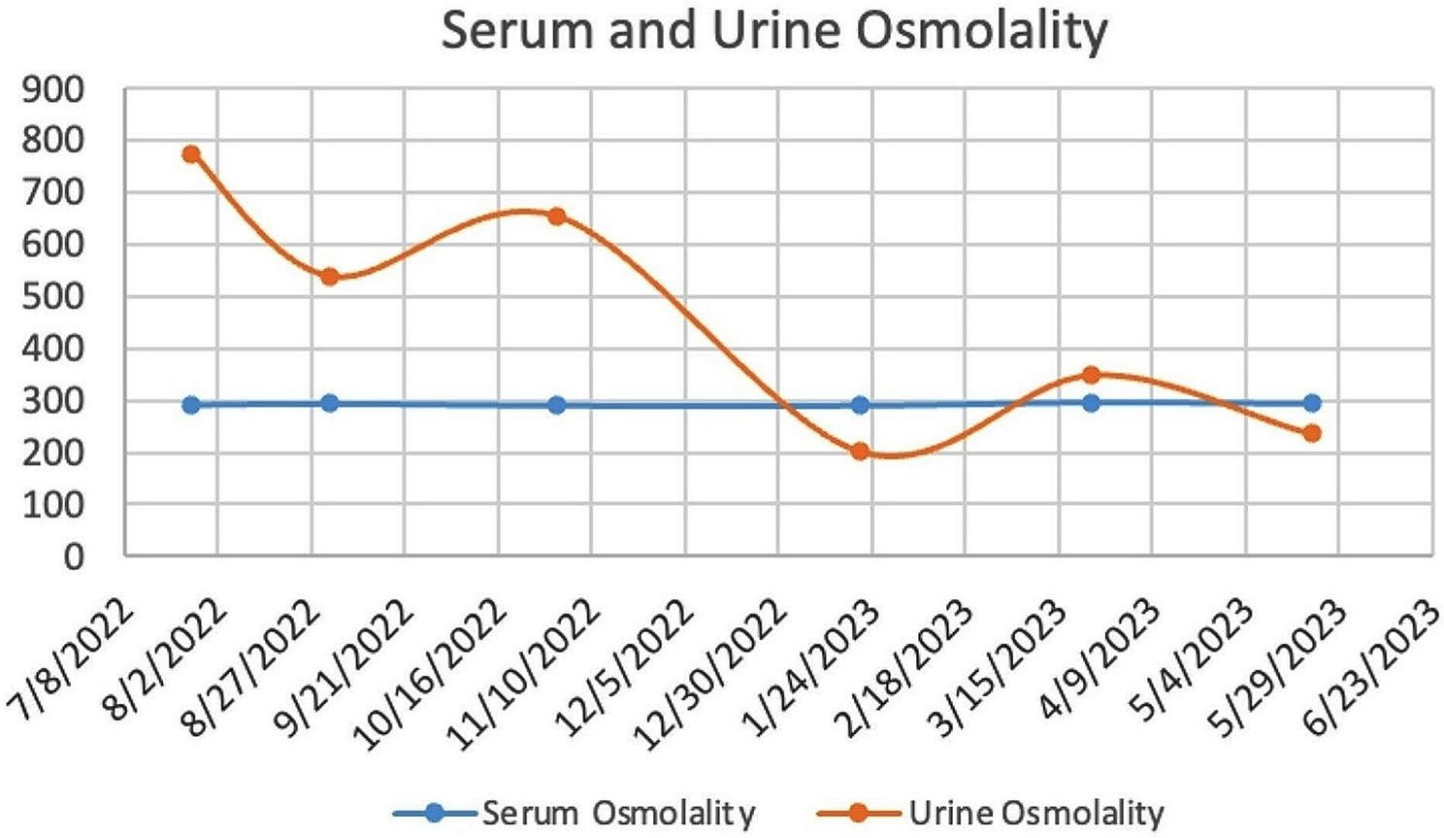
Serum and urine osmolality

She underwent a repeat endoscopic retrograde pancreatogram and sphincterotomy. Her symptoms abated following intravenous antibiotics, and her liver enzymes decreased. At the end of 2022, she developed polydipsia, polyuria, and pruritus, with frequent headaches and increasing fatigue. This necessitated her taking mid-morning and afternoon naps. She was a keen gymnast but had to cease gymnastic training due to her increasing fatigue. Investigations showed a low urinary osmolality (202mosmol/kg, *N* = 300–900) in the face of a high-normal serum osmolality of 291mosmol/kg (*N* = 275–295) (Fig. 2). Based on her serum and urine osmolality, she was diagnosed as having nephrogenic diabetes insipidus and she was commenced on hydrochlorothiazide.

**Fig. 3:**
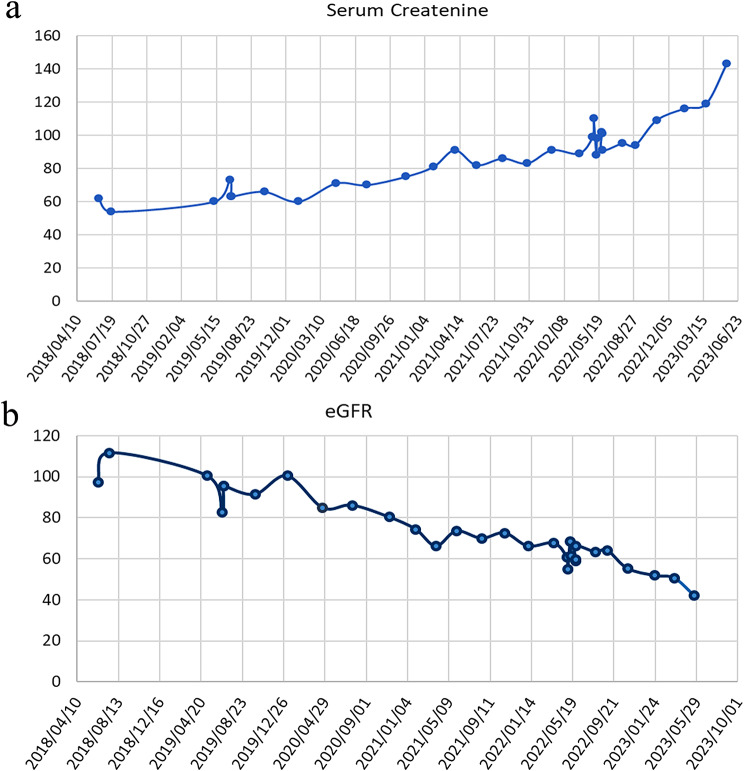
(**a**) Serum creatinine (mmol/l) (**b**) Estimated GFR (ml/min)

At her last visit there was increased proteinuria (spot urinary protein: creatinine ratio of 0.20, *N* = 0.00–0.15 g/g creatinine), raised blood urea of 8.2mmol/land creatinine of 143 umol/l (Fig. 3a), and decreased eGFR using the modified Swartz formula of 42mls/1.73m^2^/m (Fig. 3b).

Her serum bicarbonate was 20mmol/l. At her last hospital visit, she complained of non-specific symptoms of decreased appetite, pruritis and occasional headaches with painful fingers. She also complained of increased frequency of urination and polydipsia. Her clinical examination, however, was normal, with no evidence of finger swelling, visual disturbances, or any significant findings on central nervous system examination. She was commenced on levocetirizine for her pruritis and Citrosoda® (sodium bicarbonate, sodium citrate, citric acid, and tartaric acid) to correct her serum bicarbonate and hydrochlorothiazide for her diabetes insipidus. She underwent a successful live-related kidney transplant on 28 May 2024 and is presently stable. Her biological mother was the donor.

## Discussion and conclusion

We present the first confirmatory case of NPHP from South Africa based on histopathology and genetic testing. A previous report by Senior et al. from Johannesburg, South Africa, had an index patient from a family of 13 children, with four or possibly six siblings, having features of NPHP. However, NPHP was not confirmed by genetic testing as this was unavailable in 1961, and histopathological findings were reported as suggestive but inconclusive of NPHP [13]. The two methods of confirming NPHP to date are kidney biopsy or mutational analysis initiated in the context of genetic counselling.

Our patient was a Caucasian female presenting with vague abdominal pains around ten years of age, and an ultrasound showed echogenic kidneys with normal architecture. Symptoms and signs of NPHP manifest slowly and are subtle [15]. Initial imaging of patients with NPHP may be non-specific, as was the case with our patient. Ultrasound may show normal-shaped kidneys, increased echogenicity, and loss of corticomedullary differentiation. In some instances, corticomedullary cysts may be present. However, as the disease progresses, subsequent imaging at a later stage may reveal smaller, atrophic kidneys with increased echogenicity, and a more prominent cyst may develop [16].

The classic symptoms of polydipsia, polyuria, or secondary enuresis usually only begin around six years of age. If the patients have progressed to higher stages of chronic kidney disease (stage III and above), they may exhibit fatigue, pruritis, nausea, vomiting, uraemic gastritis, anaemia, and growth retardation [15]. Our patient only developed headaches, nausea, and persistent abdominal pains around 15 years of age. However, at this stage, as her sibling was diagnosed with NPHP, genetic testing was requested, confirming NPHP Type III. Two years later, as her kidney function began to deteriorate, a kidney biopsy provided further evidence of NPHP. This late manifestation of her symptoms and signs suggests she had the adolescent form of NPHP that progresses to CKF around 19 years of age. Another form of NPHP is a rare infantile form of the disease that progresses to CKF in children younger than four years [15].

Our patient’s sister had biopsy-proven liver fibrosis but no other extrarenal manifestations to suggest an associated syndrome, such as Cogan or Meckel-Gruber syndrome [11, 16]. Our patient had no extrarenal manifestations except a biochemical finding of raised liver enzymes but < 5 x normal, and the attending gastroenterologist did not undertake a liver biopsy. The differential diagnosis includes renal hypo-dysplasia, medullary cystic kidney disease complex, early onset autosomal dominant polycystic kidney disease (PKD), autosomal recessive PKD, and acquired tubulointerstitial injury. These can be distinguished by clinical, laboratory and genetic testing. Although overlap in some features can occur, genetic testing for NPHP is very specific in order to differentiate it from the conditions listed above.

As shown in Table 1, our patient carried two *NPHP3* variants in the compound heterozygosis state, consistent with the recessive inheritance of *NPHP3*. Variant c.2154 C > T, located 18 base pairs from the exon-intron boundary, previously was shown to result in the complete loss of exon 15 and to segregate with ciliopathy disease in the compound heterozygous state in three individuals in two families [17].Although ClinVar shows this variant as a Conflicting Classification of Pathogenicity, two studies, including this one, support a reclassification from likely benign/ benign to pathogenic.

Due to the high costs, genetic testing for NPHP and many other genetic diseases is unavailable in routine clinical practice in South Africa and several other low-middle-income countries. In many instances, private healthcare funders do not reimburse the cost of genetic testing. In the case of our patient and her sister, the parents paid for genetic testing privately, with some funding being raised through crowdfunding via social media. It is hoped that as the cost of genetic testing decreases, it will be more affordable in low and middle-income countries.

The current treatment options for managing NPHP include supportive care, management of complications, and kidney replacement therapy (KRT) when necessary. Supportive care involves monitoring and managing blood pressure, maintaining fluid and electrolyte balance, and addressing nutritional needs. Management of complications may include inter alia the treatment of anaemia with erythropoietin-stimulating agents and iron supplementation or the use of hypoxia-inducible factor prolyl-hydroxylase inhibitors (HIF-stabilizers), management of urinary tract infections, control of hyperphosphataemia and secondary hyperparathyroidism, correcting acid-base balance, and addressing growth and developmental issues in younger children. Additionally, extrarenal manifestations such as retinal degeneration, liver fibrosis, and skeletal abnormalities may require specialised care from relevant specialists [18]. Kidney replacement therapy (KRT) is considered when the disease progresses to CKF. Options for KRT include haemodialysis, peritoneal dialysis, and kidney transplantation. The choice of KRT depends on various factors, including the patient’s age, clinical condition, and availability of resources [18]. However, in many low-middle-income countries, access to KRT is either unavailable or limited to those patients who can afford it.

It is important to note that the specific treatment plan for NPHP should be individualised based on the patient’s clinical presentation, disease progression, and associated complications. The management approach may vary from patient to patient. Depending on organ involvement, regular follow-up visits with a nephrologist and other specialists are essential to monitor disease progression and manage complications.

Our understanding of NPHP has significantly improved from a solely histopathological entity to discovering the NPHP-causing genes and elucidating the molecular mechanisms involved. However, only 30% of patients with NPHP have an identifiable mutation, and it is possible that many more NPHP genes are still to be discovered. The identification of new genes will provide better insight into the pathophysiology of how cilia are linked to cyst formation in NPHP. A better understanding of the pathophysiology of NPHP will hopefully lead to alternative therapeutic options in addition to the conservative management of chronic kidney disease and renal replacement therapy. Although genetic testing is not readily available in many low-middle-income countries, it is hoped that more patients can be screened as this becomes more affordable.

## Data Availability

Available on request from the corresponding author.

## Abbreviations

CKF: Chronic kidney failure
HIF-stabilizers: Hypoxia-inducible factor prolyl-hydroxylase inhibitors
KRT: Kidney replacement therapy
NPHP: Nephronophthisis
US: Ultrasound

## References

[1] Luo F, Tao YH (2018). Nephronophthisis: a review of genotype–phenotype correlation. Nephrology.

[2] Ala-Mello S (1999). Nephronophthisis in Finland: epidemiology and comparison of genetically classified subgroups. Eur J Hum Genet.

[3] Macia MS (2017). Mutations in MAPKBP1 cause juvenile or late-onset cilia-independent nephronophthisis. Am J Hum Genet.

[4] Lacoste T (2007). High NPHP1 and NPHP6 mutation rate in patients with Joubert Syndrome and Nephronophthisis: potential epistatic effect of: NPHP6: and: AHI1: mutations in patients with: NPHP1: mutations. J Am Soc Nephrol.

[5] Gorden NT (2008). CC2D2A is mutated in Joubert syndrome and interacts with the ciliopathy-associated basal body protein CEP290. Am J Hum Genet.

[6] Halbritter J (2013). Defects in the IFT-B component IFT172 cause Jeune and Mainzer-Saldino syndromes in humans. Am J Hum Genet.

[7] Olbrich H (2003). Mutations in a novel gene, NPHP3, cause adolescent nephronophthisis, tapeto-retinal degeneration and hepatic fibrosis. Nat Genet.

[8] Chaki M (2011). Genotype–phenotype correlation in 440 patients with NPHP-related ciliopathies. Kidney Int.

[9] Hildebrandt F (1997). Molecular genetic identification of families with juvenile nephronophthisis type 1: rate of progression to renal failure. Kidney Int.

[10] Hildebrandt F (1992). The nephronophthisis complex: clinical and genetic aspects. Clin Investigator.

[11] Hildebrandt F, Attanasio M, Otto E (2009). Nephronophthisis: disease mechanisms of a ciliopathy. J Am Soc Nephrology: JASN.

[12] Ala-Mello S (1996). Mechanism underlying early anaemia in children with familial juvenile nephronophthisis. Pediatr Nephrol.

[13] Senior B, Friedmann A, Braudo J (1961). Juvenile familial nephropathy with tapetoretinal degeneration: a new oculorenal dystrophy. Am J Ophthalmol.

[14] Govender R, Naicker E, Pillay K (2019). A case report of a patient with Schimke Immuno-osseous dysplasia and co-morbid Moyamoya Syndrome. South Afr J Child Health.

[15] Wolf MT, Hildebrandt F (2011). Nephronophthisis Pediatr Nephrol.

[16] Smith UM (2006). The transmembrane protein meckelin (MKS3) is mutated in Meckel-Gruber syndrome and the wpk rat. Nat Genet.

[17] Molinari E (2018). Human urine-derived renal epithelial cells provide insights into kidney-specific alternate splicing variants. Eur J Hum Genet.

[18] Hildebrandt F, Zhou W (2007). Nephronophthisis-associated ciliopathies. J Am Soc Nephrol.

